# Cutaneous Small Vessel Vasculitis Associated With Varicella Zoster Infection: A Case Report and Review of the Literature

**DOI:** 10.1177/10668969251388998

**Published:** 2025-11-24

**Authors:** Josh Del Papa, Matthew J. Cecchini, A. Anurag Sharma

**Affiliations:** 1 Department of Pathology and Laboratory Medicine, 6221 Western University, London, Ontario, Canada; 2 Department of Pathology and Laboratory Medicine, London Health Sciences Centre, London, Ontario, Canada

**Keywords:** cutaneous, vasculitis, varicella zoster, infections, small vessel vasculitis, leukocytoclastic vasculitis

## Abstract

Herpes zoster is well-known for its association with large-vessel vasculitis, particularly in the central nervous system. This report highlights a rare diagnosis of small-vessel cutaneous leukocytoclastic vasculitis linked to herpes zoster. A 73-year-old man, during an extended ICU stay following an anterior myocardial infarction complicated by respiratory failure, developed a self-limiting bullous rash. Biopsy revealed epidermal infection by varicella-zoster virus (confirmed through immunohistochemistry and histologic morphology) accompanied by dermal leukocytoclastic vasculitis. This represents the 14th documented diagnosis of small-vessel leukocytoclastic vasculitis associated with varicella-zoster infection. This report broadens the spectrum of herpes zoster presentations, particularly in immunocompromised patients, and provides a valuable reference for clinicians managing atypical manifestations of this infection.

## Introduction

Varicella zoster virus (VZV) reactivation leading to herpes zoster is a common clinical concern that is typically treated with antiviral medications to prevent complications. In immunocompromised patients, especially those who have received bone marrow transplantation, reactivation presents an increased risk for localized complications and disseminated infection.^1^ In reactivation, latent virus within ganglionic neurons of the central nervous system become active as cellular immunity wanes, typically in the sixth decade of life, or in otherwise immune-compromised patients.^2^ Clinically, reactivation usually presents as herpes zoster (herpes zoster) consisting of painful lesions restricted to a single dermatome with vesicles on an erythematous base.^3^ Histologically, affected skin shows multinucleated, acantholytic keratinocytes with nuclear inclusions and perineural inflammatory infiltrate.^4^

Reactivated VZV also shows a predisposition for infection of endothelial cells, most commonly affecting the central nervous system.^5^ This generates vasculopathy which can lead to headache, cognitive impairment and focal neurological symptoms as well as ischemic stroke. Central nervous system vasculopathy associated with VZV shows a biphasic incidence in pediatric patients as well as patients over 50 years of age. Indeed, it is thought that up to 30% of ischemic strokes in pediatric patients are VZV-associated.^6^ Outside the central nervous system, VZV has been associated with large-vessel vasculitis including implications in a causative role for aortic dissection and temporal arteritis. Interestingly, it has been implied that this association is perhaps over-estimated.^7^

Very rarely, however, VZV infection has been associated with a leukocytoclastic small vessel vasculitis (LCV). LCV classically consists of an inflammatory infiltrate surrounding and disrupting vessel walls with leukocytoclasis (neutrophil degeneration) and extravasation of erythrocytes. Although frequently idiopathic, LCV has many known causative mechanisms, including systemic vasculitides autoimmune conditions, diseases of immune complex deposition, paraneoplastic syndromes, and infections (hepatitis B, hepatitis C, and syphilis among others).^8^ Although not a well-established association, to date, VZV infection has also been associated with 13 instances of cutaneous small vessel vasculitis.^9–19^ Here we present a 14^th^ report of VZV-associated cutaneous small vessel vasculitis and the most complete review of the literature to date.

## Literature Review

To contextualize our diagnosis and ensure a comprehensive comparison, we conducted a targeted literature review using PubMed and Google Scholar. Search terms included combinations of “varicella zoster,” “herpes zoster,” “leukocytoclastic vasculitis,” and “small vessel vasculitis.” We included all English-language case reports that documented cutaneous leukocytoclastic vasculitis with either clinical or histological evidence of VZV infection, confirmed by polymerase chain reaction, immunohistochemistry (IHC), culture, or histology. Exclusion criteria included reports that could not clearly differentiate between VZV and other herpesvirus etiologies. Thirteen prior patients meeting these criteria were identified and are summarized in Table 1.

**Table 1.** Prior Published Instances of VZV-Associated Cutaneous LCV.

Author (Date)	Patient Age (Sex)	Immune Status	Method of VZV Assessment
**Cohen et al** ^13^ **(1984)**	67 (male)	ICd	Culture
**Erhard et al** ^17^ **(1995)**	62 (male)	ICd	Culture and IF
**Singh and Deng** ^19^ **(1998)**	-	ICd	Culture
**Wollina et al** ^15^ **(2012)**	58 (male)	ICd	Histology
**Burgard et al** ^16^ **(2018)**	72 (male)	IM	IHC
**Clark et al** ^12^ **(2018)**	66 (male)	ICd	IHC
**Nelson et al** ^11^ **(2021)**	60 (male)	IM	IHC
**Shah et al** ^9^ **(2021)**	68 (female)	ICd	IHC
**Nastro et al** ^18^ **(2021)**	84 (female)	IM	PCR
**Afacan et al** ^10^ **(2022)**	53 (female)	ICd	Histology
**Furuoka et al** ^14^ **(2023)**	75 (male)	ICd	Histology
**Tabarsi et al** ^24^ **(2024)**	56 (male)	ICd	qPCR
**Walton et al** ^23^ **(2024)**	74 (female)	ICd	IHC
**Current study (2024)**	73 (male)	ICd	IHC

IM, immune competent; ICd, immunocompromised; IF, immunofluorescence; IHC, immunohistochemistry; PCR, polymerase chain reaction.

## Case Report

A 73-year-old man was admitted to hospital with anterior myocardial infarction. His prolonged hospital stay was complicated by respiratory failure of mixed etiology, methicillin-sensitive *Staphylococcus aureus* bacteremia, pseudomonas pneumonia, and prolonged ICU admission with multi-organ dysfunction. Approximately, 4 months following admission, the patient developed a rash following the insertion of a catheter for dialysis. The rash consisted of palpable purpura with overlying bullae over the anterior right neck and shoulder with drainage of serous fluid with pressure. At the time, this rash was thought to be related to line insertion in the context of anticoagulation as well as reactive dermatitis to the chlorhexidine applied for this procedure. It was treated empirically with hydrocortisone cream.

**Figure 1.**
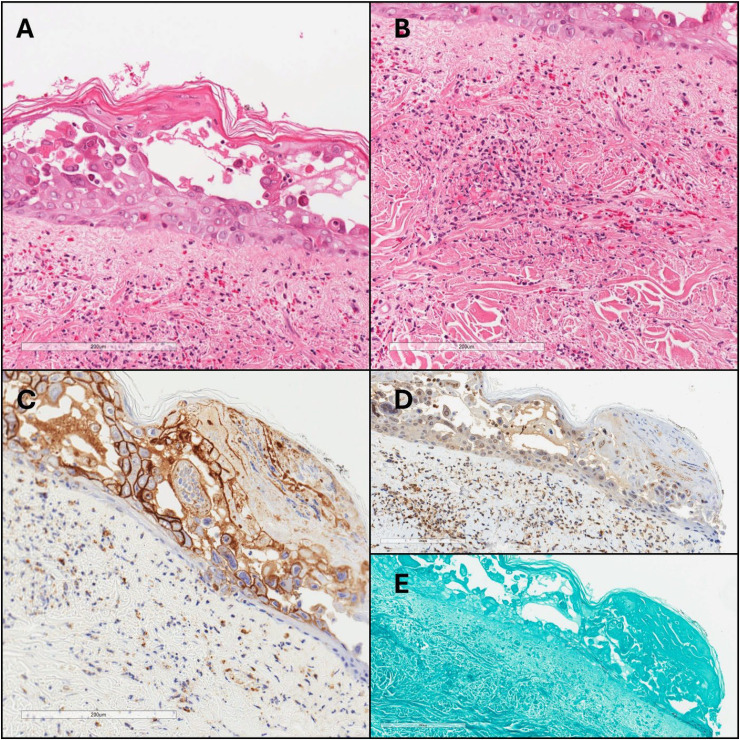
Cutaneous varicella zoster infection with underlying leukocytoclastic vasculitis. (A) Sections show acantholysis of the epidermis with the presence of multinucleated keratinocytes with nuclear margination and molding. (B) Inflammation is present within the vascular walls with leukocytoclasis and extravasation of erythrocytes. (C) Immunohistochemistry for VZV showed cytoplasmic and membranous positivity. (D) HSV1/2 and (E) GMS were negative.

A punch biopsy of the lesion (Figure 1) demonstrated acantholysis of the epidermis with the presence of multinucleated keratinocytes with nuclear margination and molding. The superficial dermis revealed inflammation of the vascular walls with leukocytoclasis and extravasation of erythrocytes, consistent with LCV. GMS stain for fungal organisms and HSV1/2 was negative. IHC for VZV showed cytoplasmic positivity.

The clinical course of this rash was unclear, however, based on available documentation, active infection was cleared without antiviral intervention by 23 days after biopsy was submitted.

## Discussion

Although VZV reactivation is well documented to induce vasculitic pathology in the CNS and in large vessels, it is not a classically established cause of small vessel cutaneous vasculitis. This study represents the 14^th^ reported such instance of a LCV associated with VZV infection. A 14^th^ report is published, however, the authors were unable to differentiate between HSV and VZV infection and thus is not included in our summary.^20^ In the present report, a 73-year-old immune-compromised man demonstrated an unexpected histological finding of VZV and concomitant LCV which was self-limiting. In the majority (11/13) of previous reports, patients were actively immune-compromised and had a mean age of 75 years (Table 1). These demographics overlap significantly with classical VZV reactivation. It is possible that LCV is more likely to occur in patients with compromised immune systems, however, these patients are also under greater scrutiny and more likely to have biopsy confirmation of unexplained rash performed.

Interestingly, in 9 out of 13 prior reports, the skin eruptions occurred on the lower extremities. On the differential diagnosis of LCV, Schamberg disease or progressive pigmented purpura, is an idiopathic phenomenon typically found in the lower extremities where osmotic pressure is increased and is characterized by leaky, fragile capillaries allowing erythrocyte extravasation which subsequently leads to an acute inflammatory response.^21^ It is possible that VZV reactivation in this area initially generates erythrocyte extravasation, which is subsequently followed by a leukocytoclastic vasculitis.

Finally, clinical herpes zoster is present in some but not all previous patients. In some instances, LCV precedes herpes zoster,^16,18^ while in others, as in ours, herpes zoster and LCV co-occur simultaneously. VZV is known to directly infect endothelial cells resulting in vasculopathy,^22^ while LCV can be triggered in many inflammatory states without direct infection of endothelial cells.^8^ It is possible that VZV-associated LCV is caused by a mixed etiology, where occasionally it is triggered by overlying herpes zoster and the resulting inflammatory state and other times triggered by direct infection of cutaneous endothelial cells by reactivated VZV.

In summary, we present the 14th reported instance of small vessel vasculitis in VZV infection. Our report is limited due to the absence of DIF, limiting our ability to confirm that herpes zoster and LCV are not simply co-occurring. Nevertheless, given most prior patients did not have a typical cutaneous VZV appearance, and in those that do, the underlying small vessel vasculitis can alter this appearance, clinicians should consider atypical VZV infection when confronted with undifferentiated rash, especially when found in immunocompromised patients. Furthermore, when presented with presumed idiopathic cutaneous LCV, IHC for VZV may help to identify the etiology and lead to earlier antiviral treatment in a subset of patients.
